# The *CsmiR397a*-*CsLAC17* module regulates lignin biosynthesis to balance the tenderness and gray blight resistance in young tea shoots

**DOI:** 10.1093/hr/uhae085

**Published:** 2024-03-28

**Authors:** Hongbin Yang, Xinyue Jia, Tong Gao, Siyu Gong, Linxuan Xia, Peiling Zhang, Yuying Qi, Shuyuan Liu, Youben Yu, Weidong Wang

**Affiliations:** College of Horticulture, Northwest A&F University, Yangling 712100, Shaanxi, China; College of Horticulture, Northwest A&F University, Yangling 712100, Shaanxi, China; College of Horticulture, Northwest A&F University, Yangling 712100, Shaanxi, China; College of Horticulture, Northwest A&F University, Yangling 712100, Shaanxi, China; College of Horticulture, Northwest A&F University, Yangling 712100, Shaanxi, China; College of Horticulture, Northwest A&F University, Yangling 712100, Shaanxi, China; College of Horticulture, Northwest A&F University, Yangling 712100, Shaanxi, China; College of Horticulture, Northwest A&F University, Yangling 712100, Shaanxi, China; College of Horticulture, Northwest A&F University, Yangling 712100, Shaanxi, China; College of Horticulture, Northwest A&F University, Yangling 712100, Shaanxi, China

## Abstract

Lignin accumulation can enhance the disease resistance of young tea shoots (*Camellia sinensis*). It also greatly reduces their tenderness, which indirectly affects the quality and yield of tea. Therefore, the regulation of lignin biosynthesis appears to be an effective way to balance tenderness and disease resistance in young tea shoots. In this study, we identified a laccase gene, *CsLAC17*, that is induced during tenderness reduction and gray blight infection in young tea shoots. Overexpression of *CsLAC17* significantly increased the lignin content in transgenic *Arabidopsis*, enhancing their resistance to gray blight and decreasing stem tenderness. In addition, we found that *CsLAC17* was negatively regulated by the upstream *CsmiR397a* by 5′-RLM-RACE, dual-luciferase assay, and transient expression in young tea shoots. Interestingly, the expression of *CsmiR397a* was inhibited during tenderness reduction and gray blight infection of young tea shoots. Overexpression of *CsmiR397a* reduced lignin accumulation, resulting in decreased resistance to gray blight and increased stem tenderness in transgenic *Arabidopsis*. Furthermore, the transient overexpression of *CsmiR397a* and *CsLAC17* in tea leaves directly confirms the function of the *CsmiR397a*-*CsLAC17* module in lignin biosynthesis and its effect on disease resistance. These results suggest that the *CsmiR397a-CsLAC17* module is involved in balancing tenderness and gray blight resistance in young tea shoots by regulating lignin biosynthesis.

## Introduction

Tea is the world’s oldest caffeinated beverage and has significant economic, medicinal, and cultural value [1]. The quality and economic value of tea are closely linked to the tenderness of the young tea shoots used to make it. Generally, the more tender the young shoots, the higher the amount of catechins, caffeine, and theanine they contain. Therefore, tea made from these shoots tends to be of higher quality and more expensive [2, 3]. However, the factors and regulatory mechanisms governing the tenderness of young tea shoots remain unclear. Lignin is closely related to the tenderness of young tea shoots and tea quality [4, 5]. As the tenderness of young tea shoots decreases and lignification increases, there is a corresponding decrease in the content of bioactive components and flavor richness of young shoots [6]. Biochemical composition analysis also confirmed a negative correlation between tenderness and lignin content in young tea shoots [7]. However, from the perspective of plant resistance, the accumulation of lignin not only enhances the sturdiness of the cell wall to prevent pathogen invasion but also inhibits the activity of fungi in infecting host cells, ultimately impeding pathogen proliferation and movement [8, 9]. Therefore, lignin may play an important role in maintaining the balance between tenderness and disease resistance in young tea shoots.

Lignin is a major component of the plant cell wall, and its biosynthetic pathways in plants have been extensively studied. Phenylalanine undergoes a series of enzymatic reactions in the cytoplasm, including deamination, hydroxylation, methylation, and reduction, resulting in the production of lignin monomers (G-/H-/S-), which are subsequently transferred to the extracellular body and catalytically polymerized to form lignin by peroxidase or laccase (LAC) in the cell wall [10]. Plant laccases, a family of polyphenol oxidases with three copper-blue structural domains, have been widely reported to mediate lignin biosynthesis, and thus participate in various life processes [11]. For example, *PlLAC4* plays a role in lignin biosynthesis in *Paeonia lactiflora* and enhances the accumulation of lignin during the thickening of secondary cell walls in the stems [12]. Disruption of *AtLAC4*, *AtLAC11*, and *AtLAC17* causes vascular and growth stagnation, the root diameter narrowing, and anther indehiscence symptoms in *Arabidopsis* due to reduced lignin accumulation [13, 14]. Similarly, silencing of *PbrLAC1*, *PbrLAC2,* and *PbrLAC18* reduced the lignin content and decreased the number of stone cells in the fruit of pear [15]. Meanwhile, numerous studies have shown that laccase mediates lignin biosynthesis to improve disease resistance in plants; for example, overexpression of *GhLac1* and *GhLac15* increases lignin accumulation to improve resistance to the fungal pathogen *Verticillium dahliae* in cotton [16, 17], and heterologous expression of eucommia *EuLac1* increases lignin content in transgenic tobacco, directly enhancing resistance to *Botrytis cinerea* [18].

MicroRNAs (miRNAs) are small 20–24 nucleotides (nt) non-coding RNAs that regulate gene expression by targeting mRNAs complementary to cleavage or translational inhibition [19]. Among, the conserved miRNA miR397 predominantly targets members of the *LAC* family in plants and plays a critical role in regulating a wide range of biological processes involved in plant growth, development, and response to external stressors [20]. For example, poplar *PtrmiR397a* regulates the expression of 17 *LACs*, leading to a reduction in lignin content [21]. *AtmiR397b* has been shown to regulate the lignin content in inflorescence stem and seed number by modulating *LAC4* expression [22] and root lignin content in response to drought and phosphorus stresses by regulating *LAC2* expression levels [23]. Similarly, the overexpression of *OsmiR397* in rice leads to a reduction in lignin deposition in the stems and the development of pre-domestication phenotypes [24]. Additionally, the *miR397-LACs* module has also been reported to be involved in the acquisition of disease resistance in plants, such as it regulates lignin accumulation in the leaves of pears and chickpea roots to protect against fungal pathogens [25, 26].

Several studies have shown that LAC-mediated lignin biosynthesis participates in plant development and stress responses, which may play an important role in the growth-defense trade-off in tea plants [27]. Recently, bioinformatics analyses predicted that *CsmiR397a* might target *CsLACs* involved in the response of tea plants to gray blight [28, 29]. Interestingly, our previous study showed that the *LAC* gene (*CsLAC17*, *CSS0040822.1*) is closely associated with changes in the tenderness of young tea shoots, which may depend on upstream *CsmiR397a* regulation [30]. The *CsmiR397a*-*CsLAC17* module appears to have significant biological functions in tenderness changes and gray blight resistance in young tea shoots. Therefore, we investigated the regulatory relationship between *CsmiR397a* and *CsLAC17*, as well as the expression levels of both in the changes of tenderness and resistance to gray blight in young tea shoots in present study. And the biological functions of *CsmiR397a* and *CsLAC17* were investigated through heterologous expression in *Arabidopsis* and native expression in tea plants. These findings will help elucidate the regulatory mechanisms of the balance between tenderness and gray blight resistance in young tea shoots, thus informing the harmonization of quality and resistance balances.

## Results

### Verification of *CsLAC17* as the target of *CsmiR397a*

**Figure 1:**
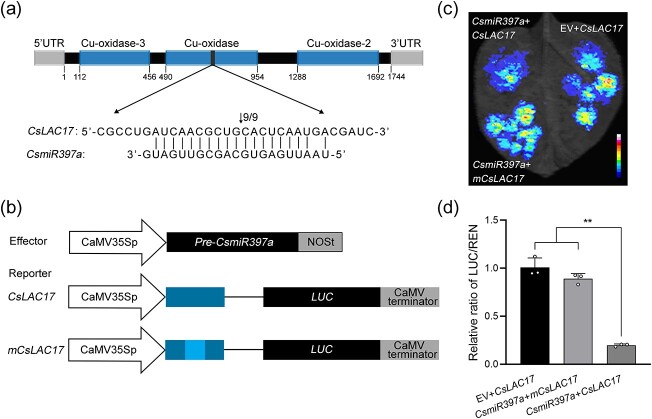
Validation of the *CsLAC17* as *CsmiR397a* target using 5′-RLM-RACE and co-transformation with *Nicotiana benthamiana*. (**a**) *CsmiR397a* cleavage site of *CsLAC17* identified using 5′-RLM-RACE. The putative cleavage site is marked with a black arrow. (**b**) Schematic representation of the constructs of the gene: Constructs of the 35 s::*Pre-CsmiR397a* used as an exogenous source of *CsmiR397a*. The reporter vectors are *CsLAC17* or m*CsLAC17* (cleavage site changed from GC to AA) fused to LUC. (**c**) Dual luciferasec complementation imaging assays. (**d**) Relative activity levels of LUC. Each bar indicates the mean ± SD from three biological replicates (^**^P < 0.01).

As shown in Fig. S1a and b (see online supplementary material), the sequence of the precursor *CsmiR397a* (*Pre-CsmiR397a*) was obtained, and it had a typical double-stranded stem-loop structure. Multiple sequence alignments demonstrated that the maturation of *miR397* in plants was highly conserved (Fig. S1c, see online supplementary material). The cDNA sequence of *CsLAC17* was cloned (Fig. S1d, see online supplementary material), and its encoded protein contained three conserved copper blue oxidation structural domains belonging to a typical LAC family member (Fig. 1a). The results of 5′-RNA ligase-mediated (RLM)-RACE showed that the cleavage site was predominantly located between the 10th and 11th nucleotides of the *miR397a-CsLAC17* matching sequence, which is in the second conserved Cu-oxidase structural domain of the *CsLAC17* mRNA sequence (Fig. 1a). To further assay targeting, the effector vector containing *CsmiR397a* and reporter vectors containing firefly luciferase (*LUC*) gene fused to *CsLAC17* or *mCsLAC17* (with the GC of the 10th and 11th bases replaced by AA) were constructed (Fig. 1b), and co-transformation of tobacco results found that co-expressed *CsmiR397a* and *CsLAC17* lowered fluorescence intensity significantly in comparison to the control (co-expressed empty vector and *CsLAC17*), but the fluorescence intensity of co-expressed *CsmiR397a* and *mCsLAC17* was similar to that of the control (Fig. 1c). LUC activity analysis showed that the co-expression of *CsmiR397a* and *CsLAC17* was significantly less active than the control and the co-expression of *mCsLAC17* (Fig. 1d). In addition, the *GUS* reporter system showed a significant decrease in the expression of the *CsLAC17*-*GUS* fusion protein due to *CsmiR397a* (Fig. S2, see online supplementary material). These findings indicate that *CsmiR397a* has a targeted cleavage effect on *CsLAC17*, thereby regulating *CsLAC17* expression.

### Expression profiles of *pre*-*CsmiR397a*, *CsmiR397a,* and *CsLAC17* during reduction of tenderness in young tea shoots

**Figure 2:**
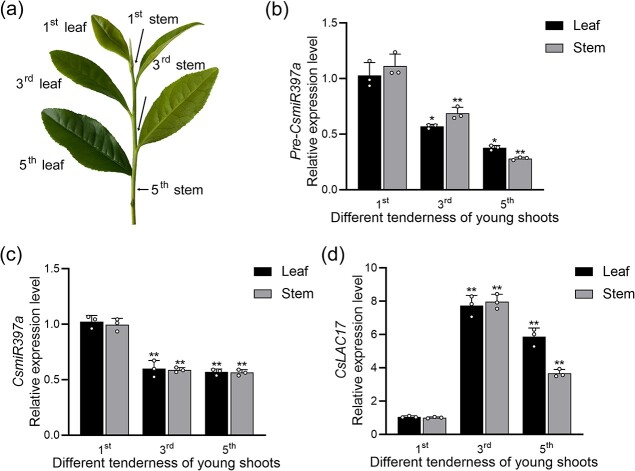
Expression profiles of *CsmiR397a*, Pre-*CsmiR397a,* and *CsLAC17* decreases during reduction in tenderness of young tea shoots. (**a**) Schematic of different tenderness of young tea shoots. (**b**, **c**, **d**) Relative expressions level of *Pre*-*CsmiR397a*, *CsmiR397a,* and *CsLAC17* at different tenderness of young tea shoots. Each bar indicates the mean ± SD calculated from three biological replicates. Asterisks indicate significant differences from the first of the same organisation, ^*^P < 0.05, ^**^P < 0.01.

Based on our previous study, the tenderness of leaves and their corresponding stems in young tea shoots diminished significantly as the position of the leaf decreased, and this process was accompanied by lignin accumulation (Fig. 2a) [30]. Here, we found that the expression level of *Pre-CsmiR397a* in the leaves and corresponding stems of young tea shoots gradually decreased as the tenderness of young tea shoots declined (Fig. 2b). The expression of *CsmiR397a* displayed a similar trend (Fig. 2c). However, the expression level of *CsLAC17* was significantly increased in the leaves and corresponding stems, demonstrating a contrasting pattern to that of *Pre-CsmiR397a* and *CsmiR397a* (Fig. 2d). These results suggest that the regulation of *CsLAC17* expression by *CsmiR397a* may be implicated in the reduction in tenderness of young tea shoots, which may mainly depend on the effect on lignin accumulation.

### Expression profiles of *pre*-*CsmiR397a*, *CsmiR397a,* and *CsLAC17* in young tea shoots response to *Pseudopestalotiopsis camelliae-sinensis* (*Ps.cs.*) infection

**Figure 3:**
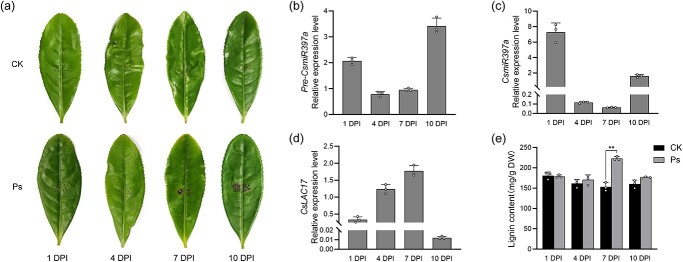
Expression profiles of *CsmiR397a*, *Pre-CsmiR397a,* and *CsLAC17* in response to *Ps.cs.* infection in young tea shoots. (**a**) Lesion development of the third leaf of tea plants inoculated with double distilled water (CK) and *Ps.cs.* DPI, days postinoculation. (**b**, **c**, **d**) Relative expression levels of *Pre-CsmiR397a*, *CsmiR397a,* and *CsLAC17* in the third leaf at differernt time points with *Ps.cs.* infection relative to double-distilled water control (CK). (**e**) Determination and analysis of lignin content in the third leaf with *Ps. camelliae-sinensis* infection relative to double-distilled water control (CK). Each bar indicates the mean ± SD calculated from three biological replicates (^**^P < 0.01).

As shown in Fig. 3a, after the inoculation of tea leaves with *Ps.cs.*, the spots progressively increased in size over time, in contrast to the control. During this process, the expression levels of both *Pre-CsmiR397a* and *CsmiR397a* first decreased and then increased (Fig. 3b and c). In contrast, the expression level of *CsLAC17* tended to increase and then decrease (Fig. 3d). Meanwhile, lignin content measurements indicated a higher value than the control in all cases after 4 d of inoculation with *Ps.cs.*, and there was a particularly significant difference, with an increase of about 40% over the control at 7 DPI (Fig. 3e). These results suggest that the reduced *CsmiR397a* expression level during the early stages of *Ps.cs.* invasion increased the expression of *CsLAC17* to promote lignin accumulation, which is essential for resisting fungal pathogen damage.

### *CsmiR397a* overexpression reduced lignin content and increased tenderness in inflorescence stem of transgenic *Arabidopsis*

**Figure 4:**
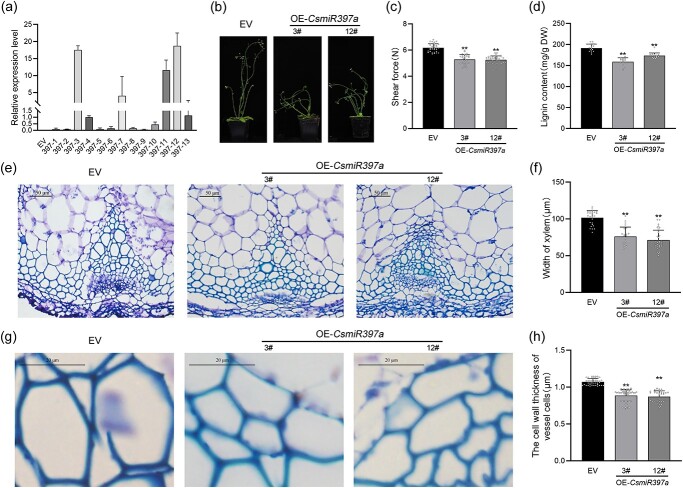
*CsmiR397a* overexpression in transgenic *Arabidopsis* plants. (**a**) Expression level of *CsmiR397a* in T2 generation transgenic plants. (**b**) *CsmiR397a*-overexpressing *Arabidopsis* exhibits greater susceptibility to collapse than *Arabidopsis* transformed with empty vector (EV). (**c**) Shear force at the base of the inflorescence stem (*n* > 20). (**d**) Total lignin content of inflorescence stems (*n* = 9). (**e**, **g**) Toluidine blue staining of transverse sections showing xylem of stem tissue in EV and two OE lines of *Arabidopsis*. (**e**) Bar = 50 μm. (**g**) Bar = 20 μm. (**f**) Xylem width in inflorescence stems (*n* > 20). (**h**) The cell wall thickness of vessel cells in the xylem of inflorescence stems (*n* > 20). Each bar indicates the mean ± SD. Asterisks indicate significant differences from EV, **P < 0.01.

Thirteen kanamycin-resistant primary transgenic plants were confirmed to contain *CsmiR397a*, and these positive plants were cultured until the T2 generation to obtain homozygous lines (Fig. S3, see online supplementary material). qRT-PCR results demonstrated that the 397a-3 and 397a-12 lines had high expression levels of *CsmiR397a*, and they were used for subsequent functional validation (Fig. 4a). Phenotypic observations showed that the inflorescence stems of the overexpressing *CsmiR397a* (OE-*CsmiR397a*) lines were more prone to lodging than the control lines transfected with the empty vector (EV), possibly because of the decreased support of inflorescence stems (Fig. 4b). Furthermore, the shear force of the inflorescence stem was significantly reduced in the OE-*CsmiR397a* lines compared to that in the EV lines (Fig. 4c), indicating that *CsmiR397a* overexpression increased the tenderness of the inflorescence stems. Lignin content was significantly reduced in the OE-*CsmiR397a* lines (Fig. 4d). In addition, the vascular tissue in the OE-*CsmiR397a* lines has fewer vessel elements and thinner xylem sections than that in the EV lines (Fig. 4e and f), and the thickness of the vessel cell walls was also significantly reduced in the OE-*CsmiR397a* lines (Fig. 4g and h). Moreover, we found that the OE-*CsmiR397a* lines displayed greater root lengths but significantly smaller leaf areas than the EV lines (Fig. S4, see online supplementary material).

### *CsLAC17* overexpression increased lignin content and reduced tenderness in transgenic *Arabidopsis*

**Figure 5:**
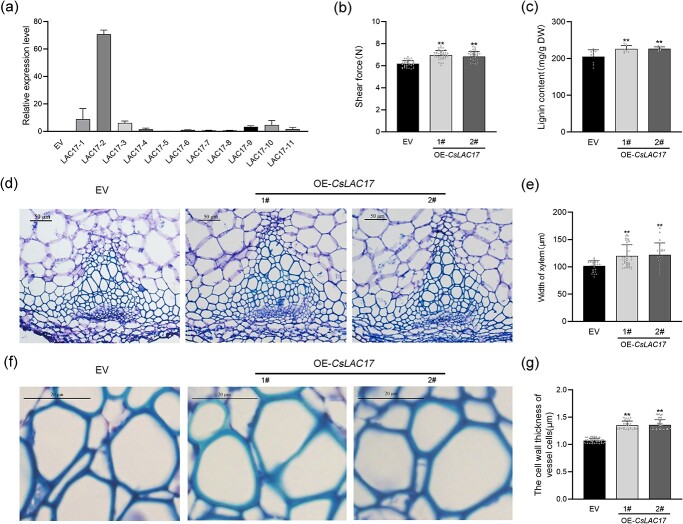
*CsLAC17* overexpression in transgenic *Arabidopsis* plants. (**a**) Expression level of *CsLAC17* in T2 generation transgenic plants. (**b**) Shear force at the base of inflorescence stems (*n* > 20). (**c**) Total lignin content of inflorescence stems (*n* = 9). (**d**, **f**) Toluidine blue staining of transverse sections showing xylem of stem tissue in EV and two OE lines of *Arabidopsis*. (**d**) Bar = 50 μm. (**f**) Bar = 20 μm. (**e**) Xylem width of inflorescence stems (*n* > 20). (**g**) The cell wall thickness of vessel cells in the xylem of inflorescence stems (*n* > 20). Each bar indicates the mean ± SD. Asterisks indicate significant differences from EV, **P < 0.01.

Eleven kanamycin-resistant primary transgenic plants were confirmed to contain *CsLAC17* (Fig. S5, see online supplementary material), and two transgenic lines with high levels of *CsLAC17* expression (*LAC17*–1 and *LAC17*–2) were selected for functional validation (Fig. 5a). The shear force of the inflorescence stems showed a significant increase in the overexpressing *CsLAC17* (OE-*CsLAC17*) lines compared to that in the EV lines (Fig. 5b), implying that overexpression of *CsLAC17* decreased the tenderness of the inflorescence stems. Similarly, lignin content was significantly increased in the OE-*CsLAC17* lines (Fig. 5c). In addition, the vascular tissue in the OE-*CsLAC17* lines has more vessel elements and wider xylem sections than that in the EV lines (Fig. 5d and e), and the thickness of the vessel cell walls was also significantly increased in the OE-*CsLAC17* lines (Fig. 5f and g). The OE-*CsLAC17* lines showed a significant increase in root length compared to the EV lines (Fig. S6, see online supplementary material), except for other phenotypes that were not significantly different.

### Analysis of disease resistance in transgenic *Arabidopsis*

**Figure 6:**
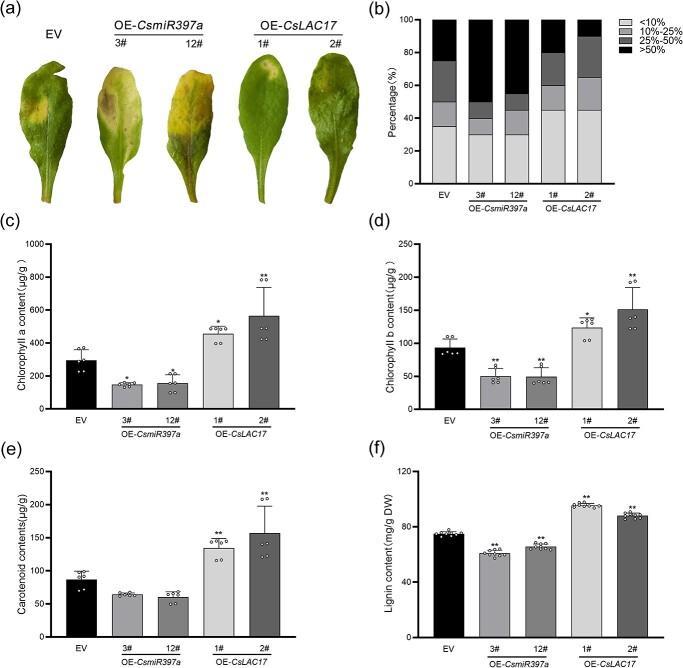
Transgenic *Arabidopsis* leaf with *Ps.cs.* infection. (**a**) Photographs of representative leaves 72 h after infection with *Ps.cs*. (**b**) Percentage of leaves with four levels of disease progression according to the size of the lesion area relative to the half of leaf 72 h after infection with *Ps.cs.* (**c**–**e**) Chlorophyll a, chlorophyll b, and carotenoid content of leaves 72 h after infection with *Ps.cs.* (*n* = 6). (**f**) Total lignin content of transgenic *Arabidopsis* leaves (*n* = 9). Each bar indicates the mean ± SD. Asterisks indicate significant differences from EV, *P < 0.05, **P < 0.01.

To investigate the effect of overexpression of *CsmiR397a* and *CsLAC17* on disease resistance in *Arabidopsis*, the degree of disease was evaluated 3 d after the leaves had been infected with *Ps.cs*. The results revealed that the OE-*CsmiR397a* lines caused more severe damage than the EV lines, with the majority of the leaves yellowing and losing their green color, whereas the OE-*CsLAC17* lines displayed a dissimilar phenotypic profile with less leaf yellowing and greening, which was significantly lower than that of the control lines (Fig. 6a). The degree of incidence was classified into four levels based on the green loss as a percentage of the half-leaf area: level 1 (<10%), level 2 (10–25%), level 3 (25–50%), and level 4 (>50%). Statistical analysis indicated that the OE-*CsmiR397a* lines were more at level 4 and less at level 1, whereas the OE-*CsLAC17* lines were more at level 1 and less at level 4, with less severe damage than the EV lines (Fig. 6b). Correspondingly, both chlorophyll a and b content were significantly lower in the leaves of the OE-*CsmiR397a* lines than in those of the EV lines after *Ps.cs.* infection, whereas they were significantly higher in the leaves of the OE-*CsLAC17* lines than in those of the EV lines (Fig. 6c and d). In contrast, the carotenoid content was significantly increased only in the leaves of the OE-*CsLAC17* lines, but the difference in the leaves of the OE-*CsmiR397a* lines was not significant, although it was lower than that in the EV lines (Fig. 6e). In addition, the analysis of lignin content showed an increase of about 27% and 17% in the leaves of the OE-*CsLAC17* lines compared to that in the leaves of the EV lines (Fig. 6f). In contrast, the lignin content in the leaves of OE-*CsmiR397a* lines decreased by about 20% and 13%, respectively (Fig. 6f). These findings indicate that *CsmiR397a* and *CsLAC17* affected *Arabidopsis* leaf resistance to *Ps.cs.* by regulating the biosynthesis and accumulation of lignin.

### Validation of the regulation of *CsLAC17* by *CsmiR397a* in tea plants

**Figure 7:**
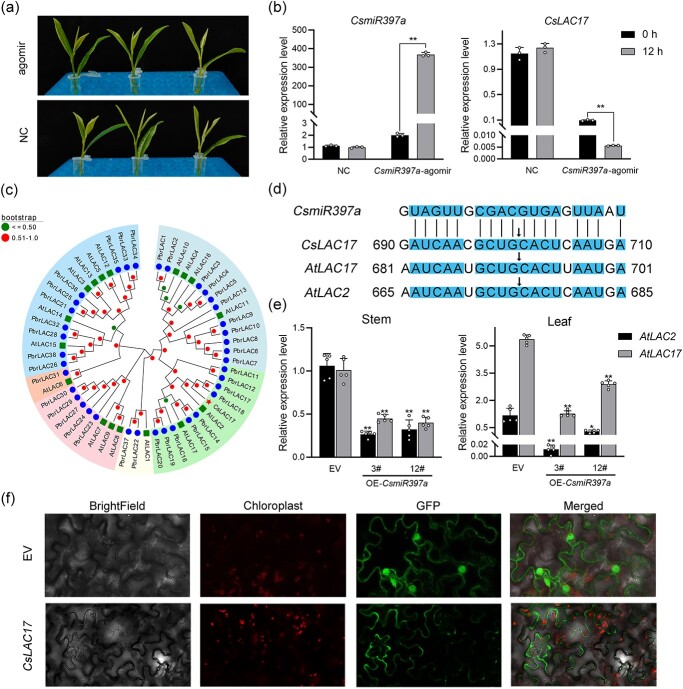
Validation of the regulation of *CsLAC17* by *CsmiR397a* in tea plants. (**a**) Schematic of *CsmiR397a*-agomir treatment. (**b**) Relative expression levels of *CsmiR397a* and *CsLAC17* by *CsmiR397a*-agomir treatment in tea plants (*n* = 3). (**c**) Phylogenetic relationship of *CsLAC17*, 38 *PbrLACs,* and 17 *AtLACs*. (**d**) Similarity analysis of *CsmiR397a* cleavage sites of *CsLAC17*, *AtLAC17,* and *AtLAC2*. (**e**) Relative expression levels of *AtLAC2* and *AtLAC17* in leaves and stems of *CsmiR397a*-overexpressing *Arabidopsis* (*n* = 5). (**f**) Subcellular localization of CsLAC17. Each bar indicates the mean ± SD. Asterisks indicate significant differences from EV, *P < 0.05, **P < 0.01.

To verify the regulatory effect of *CsmiR397a* on *CsLAC17* expression in tea plants, the miRNA-agomir method was used to transiently overexpress *CsmiR397a* in the tender shoots of tea plants (Fig. 7a). The results showed that the expression of *CsmiR397a* increased significantly after 12 h of miRNA-agomir treatment, whereas that of *CsLAC17* decreased significantly (Fig. 7b). Phylogenetic analysis revealed that *CsLAC17* clustered with *AtLAC2* and *AtLAC17*, indicating that they were highly homologous (Fig. 7c). A comparison with the studied *miR397a* targeting complementary sequences in *Arabidopsis* demonstrated that *CsLAC17* was highly conserved with *AtLAC2* and *AtLAC17*, including identical cleavage sites (Fig. 7d). Furthermore, qRT-PCR confirmed that the overexpression of *CsmiR397a* significantly repressed the expression of *AtLAC2* and *AtLAC17* (Fig. 7e). These results confirmed the regulatory effect of *CsmiR397a* on *CsLAC17* and implied that the *CsmiR397a*-*CsLAC17* module had a similar biological function in both transgenic *Arabidopsis* and tea plants. Furthermore, subcellular localization analysis revealed that *CsLAC17* was localized in the cell wall, supporting its role in mediating the biological function of lignin biosynthesis (Fig. 7f).

### The *CsmiR397a*-*CsLAC17* module regulates lignin biosynthesis to influence the disease resistance in tea plants

**Figure 8:**
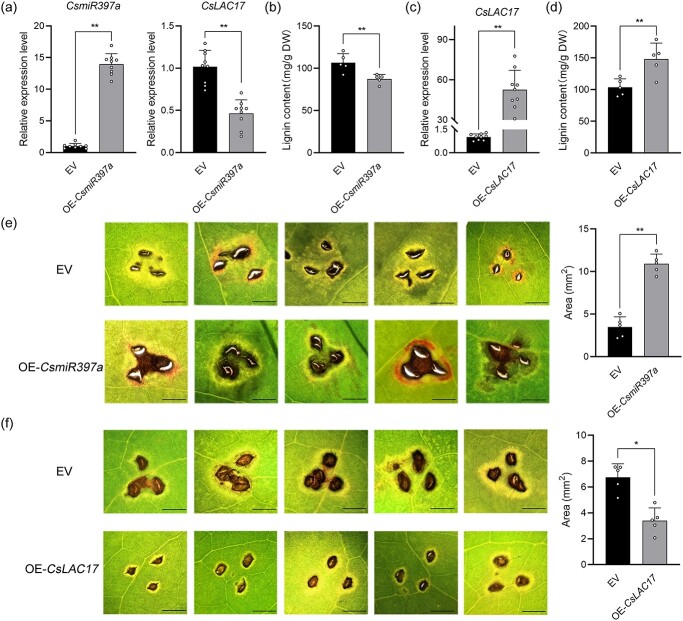
Functional verification of the *CsmiR397a-CsLAC17* module in tea plants. (**a**) Relative expression levels of *CsmiR397a* and *CsLAC17* in OE-*CsmiR397a* and EV leaves (*n* = 9). (**b**) Total lignin content in OE-*CsmiR397a* and EV leaves (*n* = 5). (**c**) Relative expression levels of *CsLAC17* in OE-*CsLAC17* and EV leaves (*n* = 9). (**d**) Total lignin content in OE-*CsLAC17* and EV leaves (*n* = 5). (**e**) Disease symptoms of OE-*CsmiR397a* and EV leaves 5 days after infection with *Ps.cs.* (*n* = 5), Bar = 2.5 mm. (**f**) Disease symptoms of OE-*CsLAC17* and EV leaves 7 days after infection with *Ps.cs.* (n = 5), Bar = 2.5 mm. Each bar indicates the mean ± SD. Asterisks indicate significant differences from EV, *P < 0.05, **P < 0.01.

To investigate the role of the *CsmiR397a*-*CsLAC17* module in lignin biosynthesis in tea plants, the transient transformation validation was carried out (Fig. S7, see online supplementary material), and the results revealed that the expression level of *CsmiR397a* was significantly increased, while the expression level of its target gene *CsLAC17* was significantly decreased in the leaves overexpressing *CsmiR397a* (Fig. 8a). Meanwhile, the lignin content in OE-*CsmiR397a* leaves was reduced by about 18% compared to that in the EV leaves (Fig. 8b). In contrast, overexpressing *CsLAC17* significantly increased the expression of *CsLAC17* and enhanced the content of lignin in the leaves of tea plant (Fig. 8c and d). In addition, the results of *Ps.cs*. infection showed that the leaves overexpressing *CsmiR397a* exhibited a larger lesion area than the EV leaves (Fig. 8e). Conversely, the leaves overexpressing *CsLAC17* showed a smaller lesion area than the EV leaves (Fig. 8f). These results suggest that the *CsmiR397a*-*CsLAC17* module is involved in regulating lignin biosynthesis in young tea shoots, which is crucial for the acquisition of disease resistance in tea plants.

## Discussion

Young tea shoots have been used for tea production for thousands of years, and their tenderness is an important factor in tea quality and yield [31]. Therefore, it is essential to understand the regulatory mechanisms underlying young shoots changes in tea plants. Our previous study showed that lignin accumulation leads to a reduction in the tenderness of young tea shoots and that the involvement of *LAC* family members linked to lignin biosynthesis plays an important role in this process [30]. Here, we found that the expression of *CsLAC17*, a typical member of the *LAC* family, significantly increased with decreased tenderness in young tea shoots, hypothesizing that it might be involved in lignin biosynthesis in the process of tenderness decrease in young tea shoots. Multiple studies have indicated that the later stages of plant lignin synthesis predominantly occur in the cell wall, and LAC has been shown to be predominantly localized in the cell wall. For example, CsiLAC17 is localized in the cell wall and is involved in lignin synthesis [32]; PbrLAC1, PbrLAC2, and PbrLAC18, which play a role in the accumulation of lignin, were also localized in the cell wall [15]. Unsurprisingly, CsLAC17 is also a cell wall-localized protein, implying that it functions in lignin biosynthesis. Meanwhile, transgenic *Arabidopsis* overexpressing *CsLAC17* showed a significant increase in lignin content, which led to a heightened lignification process in the inflorescence stems. Similarly, transient overexpression of *CsLAC17* also significantly increased the lignin content in tea leaves, directly confirming the biological function of *CsLAC17* in lignin biosynthesis. In addition, analysis of shear force to quantify tenderness revealed that the accumulation of lignin in overexpressing *CsLAC17 Arabidopsis* also resulted in reduced tenderness. These results suggested that *CsLAC17* dominated lignin synthesis and accumulation, subsequently decreasing the tenderness of young tea shoots and transgenic *Arabidopsis* inflorescence stems.

Gray blight is a highly destructive fungal leaf disease in tea plants that can cause significant leaf shedding and restrict tea plant growth, resulting in reduced yield and quality [33]. Simultaneously, the application of chemical fungicides that target gray blight frequently presents a substantial risk to the safety of tea products [34]. Therefore, enhancing the resistance of tea plants is a fundamental way to reduce the damage caused by pathogens. Numerous studies have confirmed that an increase in lignin levels in plants provides broad-spectrum resistance to pathogens, and that LAC is crucial. For example, *LAC4* enhances resistance to *V. dahlia* by increasing lignin accumulation in cotton [35]. Similarly, *LAC* promotes lignification of apple roots to protect against *Pythium ultimum* infection [36]. In the present study, *CsLAC17* expression was induced by gray blight infection, particularly during the pre-infection period, which is consistent with the induction of *LACs* in tea plants by gray blight, as reported by Zheng *et al.* [37], indicating that *CsLAC17* is involved in the response of tea plants to gray blight. Our findings confirm that these processes are accompanied by lignin accumulation. Combined with previous studies, it is reasonable to speculate that the heightened expression of *CsLAC17* promotes lignin accumulation in response to gray blight infection. This inference was confirmed by the overexpression of *CsLAC17* in *Arabidopsis* and tea plants. Specifically, the leaves of overexpressed *CsLAC17* plants demonstrated increased resistance to *Ps.cs.*, including a smaller spot area and richer cytochromes. The above-mentioned enhancement of disease resistance depends on the accumulation of lignin, which is similar to that reported by Yu *et al.* [38]. These results indicate that the dominance of *CsLAC17* in lignin biosynthesis and accumulation is essential for improving resistance to gray blight, which is observed not only in transgenic *Arabidopsis* but also in tea plants.

An increasing number of studies have shown that *miR397* targets and regulates the expression of the LAC family genes, thereby influencing lignin accumulation and participating in many plant processes. For example, *AtmiR397* affects cadmium tolerance in *Arabidopsis* by regulating the expression of *AtLAC*2/4/17 and changing lignin content [39]; and heterologous expression of *SvmiR397* in *Arabidopsis* resulted in decreased expression of three *LAC* genes, causing a reduction in lignin content and an increase in sensitivity to salt stress [40]. Our findings demonstrate that the *CsLAC17* sequence contained a conserved cleavage site for *miR397a* and its expression was regulated by *CsmiR397a* targeting, which confirms the speculation of Zhu *et al.* [28]. Meanwhile, the lignin content was significantly reduced in overexpressing *CsmiR397a Arabidopsis* as expected, and this change led to increased tenderness of the inflorescence stem, as evidenced by a significant reduction in mechanical support and shear force. Moreover, the transgenic *Arabidopsis* exhibited reduced resistance to pathogens, as evidenced by larger spot areas and a greater loss of green color. Interestingly, the expression levels of *AtLAC2* and *AtLAC17*, which are highly conserved homologues of *CsLAC17*, were significantly decreased in overexpressing *CsmiR397a Arabidopsis*. Additionally, the regulation of *CsLAC17* by *CsmiR397a* was confirmed in tea plants using the miRNA-agomir and transient transformation technology. Among, overexpressing *CsmiR397a* significantly reduced the lignin content in the leaves of tea plant, and this also led to the weakening of disease resistance. Therefore, the *CsmiR397a*-*CsLAC17* module affects the biosynthesis and accumulation of lignin, crucially contributing to the changes in tenderness and development of resistance to gray blight in young tea shoots.

**Figure 9:**
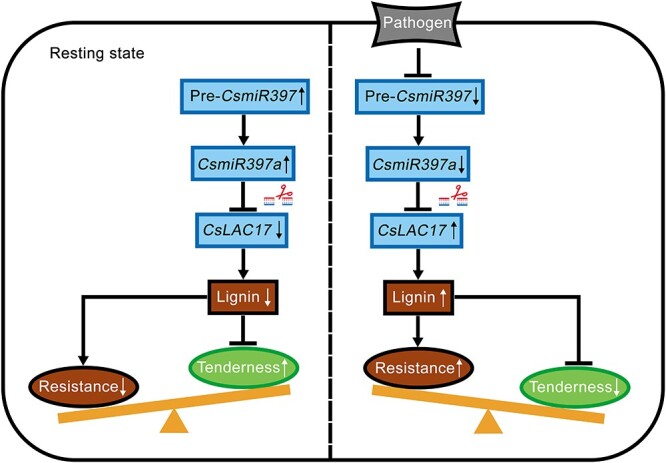
The *CsmiR397a*-*CsLAC17* module regulates lignin biosynthesis to balance tenderness and resistance in young tea shoots. *CsmiR397a* acts as a negative posttranscriptional regulator of *CsLAC17*. In the resting state, upregulation of *CsmiR397a* leads to downregulation of *CsLAC17* participated in lignin biosynthesis to further enhance the tenderness and weaken the resistance. In contrast, pathogen-induced downregulation of *CsmiR397a* promotes the expression of *CsLAC17*, leading to a rapid accumulation of lignin, which increases the resistance but reduces the tenderness.

It is well known that elevations in plant defense typically occur at the expense of other processes, such as growth retardation, yield, and quality reduction [41]. This tradeoff between growth and defense poses great difficulties for crop genetic improvement [42]. Over the past decade, some progress has been made in understanding the genes, pathways and regulatory networks involved in the ‘growth-defense’ trade-off. For example, the balance between disease resistance and rice yield is controlled by the regulation of *OsWRKY45* [43]. Similarly, rapamycin kinase modulates salicylic acid and jasmonic acid levels to regulate growth-defense trade-offs in plants [44]. Our study also showed that the accumulation of lignin reduced the tenderness of young tea shoots but enhanced the resistance to gray blight. The *CsmiR397a*-*CsLAC17* module played an important role in this process (Fig. 9). Specifically, high expression of *CsmiR397a* inhibited the expression of *CsLAC17* to reduce lignin accumulation, thus preserving the tenderness of young tea shoots. In contrast, decreased expression of *CsmiR397a* led to increased expression of *CsLAC17*, promoting lignin accumulation and enhancing disease resistance, but diminishing tenderness. Therefore, achieving a balance between tenderness and resistance through the *CsmiR397a*-*CsLAC17* module is important for coordinating the yield, quality, and resistance of tea plants, as well as for selecting superior tea plant cultivars.

## Materials and methods

### Plant materials and pathogen infection

The ‘Zhongcha 108’ cultivar tea plant (*Camellia sinensis*) was grown at Tea Test and Demonstration Station of Northwest A&F University, and some healthy ones with similar growth were selected for use. The pathogen *Pseudopestalotiopsis camelliae-sinensis* (*Ps.cs*.) has been isolated prior from tea gray blight, and 50 μL spore suspension (10^7^ conidia per mL) was applied to the third leaf that had been injured. Third leaves treated with sterile distilled water of the same volume were used for the control. Then, the leaf samples were observed and collected at 1, 4, 7, and 10 days (DPI) in three independent biological replicates. *Arabidopsis* was grown in nutrient soil at 25°C, under 60% humidity and a 16 h/8 h (light/darkness) photoperiod. Infestation of 15 μL spore suspension with same concentration was performed on 7-week-old leaves of transgenic *Arabidopsis*.

### Cloning and characterization analysis of *CsmiR397a* and *CsLAC17*

Genomic DNA extracted by the CTAB method [45] was used to clone the DNA fragment containing the precursor *CsmiR397a* (*Pre-CsmiR397a*) and its flanking regions, and the specific primers used are listed in Table S1 (see online supplementary material). The online software RNAfold web server and ClustalX were used for secondary structure predictions and multiple sequence comparisons. Total RNA was extracted using a SteadyPure Plant RNA Extraction Kit (Accurate, Changsha, China), and cDNA was generated using an Evo M-MLV Plus 1st Strand cDNA Synthesis Kit (Accurate, Changsha, China). The coding sequence of *CsLAC17* was amplified using cDNA as a template with the specific primers (Table S1, see online supplementary material). The sequence of *CsLAC17* was analysed using the NCBI website, and then constructed into a phylogenetic tree with 38 pear *LAC* genes and 17 *Arabidopsis LAC* genes using MEGA software with the neighbor-joining method. Multiple sequence alignment was performed using DNAMAN software.

### Computational prediction and experimental validation of *CsmiR397a* target genes

The cleavage site of *CsLAC17* was predicted using the psRNATarget tool. Experimental validation of predicted targets was performed by 5′-RLM-RACE verification of miRNA cleavage sites in target genes using the RLM-RACE kit (Thermo, New York, USA) as previously described [46]. Then, PCR products were gel-purified and cloned into the pCE2 vector (Vazyme, Nanjing, China), and nine independent clones were sequenced.

### Dual luciferase assay in *N. benthamiana* leaves

The dual-luciferase assay was carried out according to a previously described method with minor modifications [15]. For this evaluation, one effector and two reporters containing 35S::*CsmiR397a*, 35S::*CsLAC17*::LUC, and 35S::m*CsLAC17*::LUC were compared. The mutant sequence, *mCsLAC17*, is a change of two bases (GC to AA) at the cleavage site of the target sequence. Renilla luciferase (REN) was present in the same vector as LUC and served as an internal control for the normalization of LUC expression. *Agrobacterium tumefaciens* (GV3101) containing effector or reporter vectors were cultured individually until an OD_600_ of 0.8–1.0 was reached. Subsequently, they were resuspended with MES buffer and co-transformed into *Nicotiana benthamiana* leaves. After 2 d of incubation, observations were made using CDD (Princeton Instruments, USA). The activity of LUC and REN were determined using the Dual-Luciferase Reporter Kit (Transgen, Beijing, China), and the relative ratio of LUC/REN was calculated. Each experiment included three biological replicates of each treatment group. All the primers used are listed in Table S1 (see online supplementary material).

### Regulatory function verification of *CsmiR397a* in tea plants through RNA oligonucleotide treatment

To investigate the regulatory function of *CsmiR397a* in tea plants, the artificial synthesized *miRNA* was transferred into the tender shoots of tea plants as previously described [47]. Specifically, newly harvested young shoots were immediately placed in the solution containing 20 μM *CsmiR397a*-agomir (miRNA overexpression) or its negative control, respectively. And then, the leaf samples were collected for qRT-PCR analysis after 12 h. The details of the RNA oligonucleotides are listed in Table S1 (see online supplementary material).

### qRT-PCR analysis

The first-strand cDNA for miRNA quantification was synthesized by a miRNA 1st strand cDNA synthesis kit (Accurate, Changsha, China). Subsequently, qRT-PCR was performed using a LightCycler 480 (Roche, Basel, Switzerland). Relative expression levels of all genes were computed employing the 2^-ΔΔCt^ algorithm, with *CsActin* and *CsmiR222* serving as reference genes for *CsLAC17* and *CsmiR397a*, respectively [46]. The reference genes for *AtLAC2*, *AtLAC17*, and *AtmiR397a* were *AtActin* and *AtU6*. Each gene was repeated for at least three biological samples and all the specific primers used are listed in Table S1 (see online supplementary material).

### *Arabidopsis* plant transformation

*Pre-CsmiR397a* and *CsLAC17* were cloned into the binary vector pCAMBIA2300-GFP containing the CaMV35S promoter for 35S::Pre-CsmiR397a and 35S::CsLAC17::GFP vector construction. All resulting plasmids and empty vector (EV) were chemically transformed into *A. tumefaciens* GV3101. The *Arabidopsis* plants were transformed using the flower-dip method [48]. T2 and T3 homozygous lines were used for all experiments presented in this study. All the primers used are shown in Table S1 (see online supplementary material).

### Subcellular localization analysis

The 35S::CsLAC17::GFP vector and EV were transformed into *A. tumefaciens* GV3101. Subsequently, they were transiently transformed into 6-week-old *N. benthamiana* leaves and incubated at 24°C with a 16 h/8 h (light/darkness) photoperiod for 2–3 days. Then, the transformed leaves were collected and observed for fluorescence signals by a laser scanning confocal microscope (Leica, Germany) according to previous descriptions [45].

### *A. tumefaciens*-mediated transient overexpression in tea leaves

Transient transformation of the target genes into tea leaves was performed according to a previous method [49]. *A. tumefaciens* transformed with the *CsLAC17* and *Pre-CsmiR397a* construct was grown in liquid LB medium, and then they were centrifugally collected and resuspended to a final OD_600_ of 1.0 for injection. *A. tumefaciens* containing the EV with the GFP tag was injected as a control. Leaf samples were collected 4 days after injection, and used for further gene expression analysis, lignin content determination, and disease resistance evaluation.

### Histological microscopic analysis

Inflorescence stem bases of 7-week-old transgenic *Arabidopsis* were harvested and treated with FAA fixative for more than 24 h. Samples were dehydrated using an ethanol series and embedded in paraffin. Cross-sections (10 μm) were cut using a Leica RM 2015 ultramicrotome (Leica, Germany). The sections were mounted on slides and stained with 1% (w/v) toluidine blue staining solution (containing 0.7% (v/v) ethanol) for 10 min. Subsequently, the sections were examined using a fully automated upright fluorescence microscope (Olympus, Japan), and the xylem width and cell wall thickness of the inflorescence stems was measured. The xylem width and cell wall thickness of each line was determined by averaging the measurements of six sections at the diagonal position of the stem cross-sections of five plants in each line.

### Determination of shear force

Maximum shear was used to quantify changes in the tenderness of transgenic *Arabidopsis* plants as follows: seven-week-old transgenic *Arabidopsis* inflorescence stems were harvested at the base and placed perpendicular to the leaf on the bench of a TMS pilot texture analyser (FTC Corporation, USA). Subsequently, 90% of the stem was vertically sheared. The working curve of the maximum shear force was recorded and each set of samples from the five biological replicates was repeated at least 10 times.

### Analysis of lignin content

For the determination of lignin content, the plant tissues, such as the leaves and inflorescence stems of the transgenic *Arabidopsis* and the leaves and stems of tea plant, were collected and stored at −80°C after drying. The lignin content was determined according to previously established protocols [50]. The commercial alkaline lignin (Sigma-Aldrich, USA) was used to construct a linear calibration curve.

### Determination of chlorophyll content

Briefly, 0.1 g of disease-infected *Arabidopsis* leaves was thoroughly homogenized with an appropriate volume of 95% ethanol under dark conditions at 4°C. The absorption intensities of light for chlorophyll a and b at 663 nm and 646 nm and for carotenoids at 470 nm were measured. The contents of chlorophyll a and b, as well as carotenoids, were calculated using the equations described by predecessors [39].

### Statistical analysis

To conduct statistical analyses, GraphPad Prism 9.0 software was used. Differences between means were compared by *t*-test and analysis of variance (ANOVA).

## Supplementary Material

Web_Material_uhae085

## Data Availability

All relevant data can be found within the paper and its supporting materials.
