# Supplementary material for: The CsmiR397a-CsLAC17 module regulates lignin biosynthesis to balance the tenderness and gray blight resistance in young tea shoots
Source: Hortic Res. 2024 Mar 28;11(5):uhae085. doi: 10.1093/hr/uhae085 (PMC11116903; doi:10.1093/hr/uhae085)
Supplement: Web_Material_uhae085 [file web_material_uhae085.zip › Supporting Information-R1.docx]

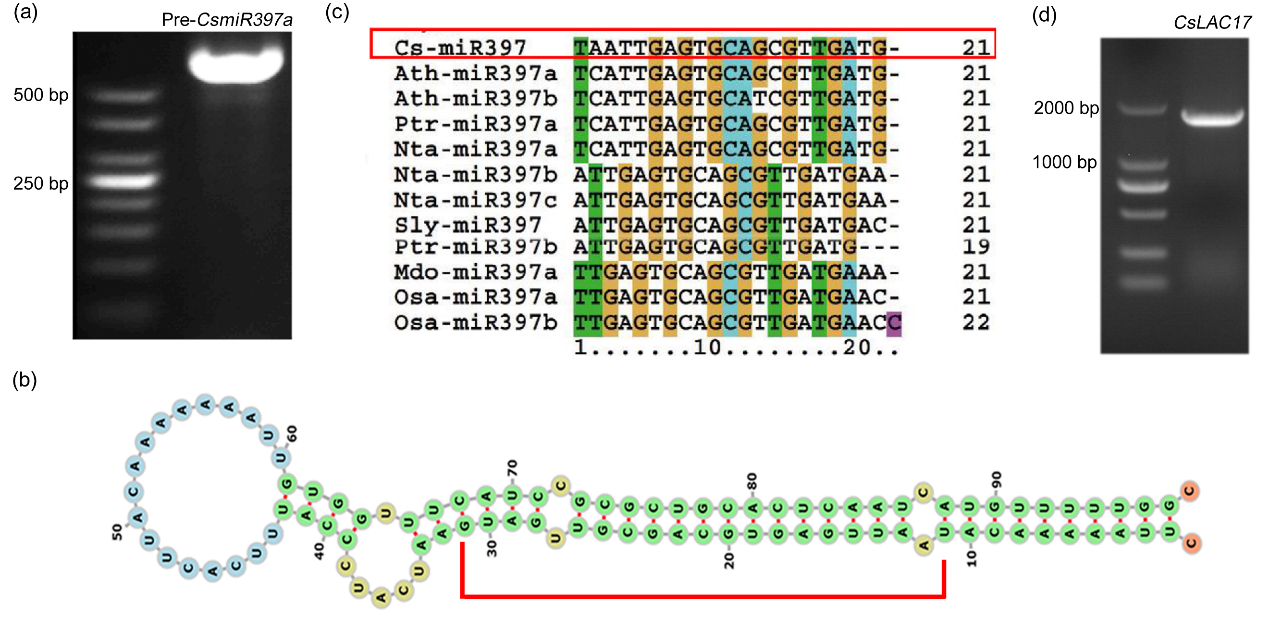


**Figure S1.** Cloning and bioinformatics analysis of *CsmiR397a* and *CsLAC17*. (a) Cloning of precursor *CsmiR397a* sequence. (b) *CsmiR397a* secondary structure prediction. (c) Multiple sequence comparison of *CsmiR397a* mature bodies. (d) Cloning of *CsLAC17* sequence.


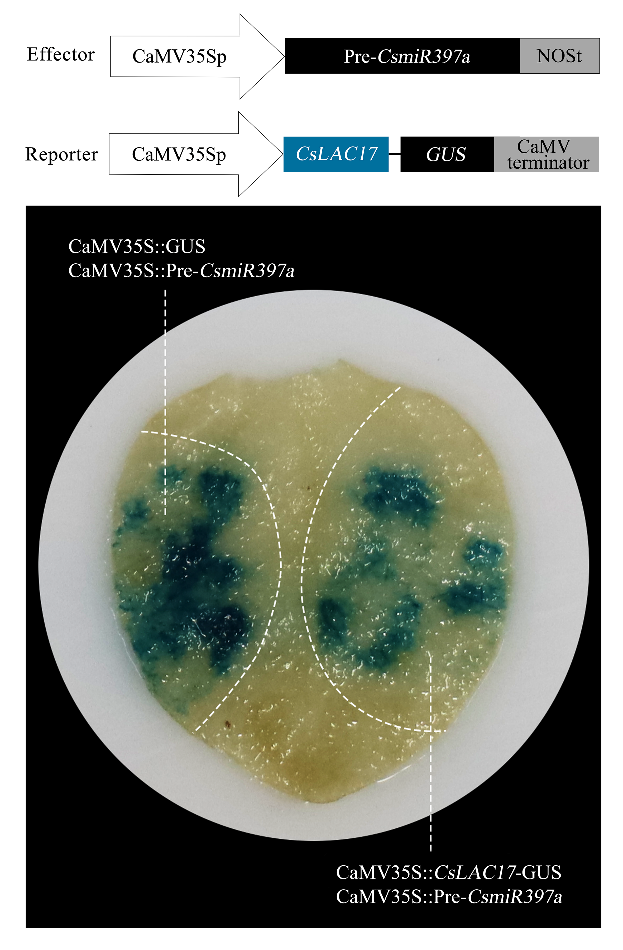


**Figure S2.** β-glucuronidase (GUS) staining in N. benthamiana co-transformation assays to analyze the regulatory role of *CsmiR397a* in repressing the expression of the *CsLAC17*.
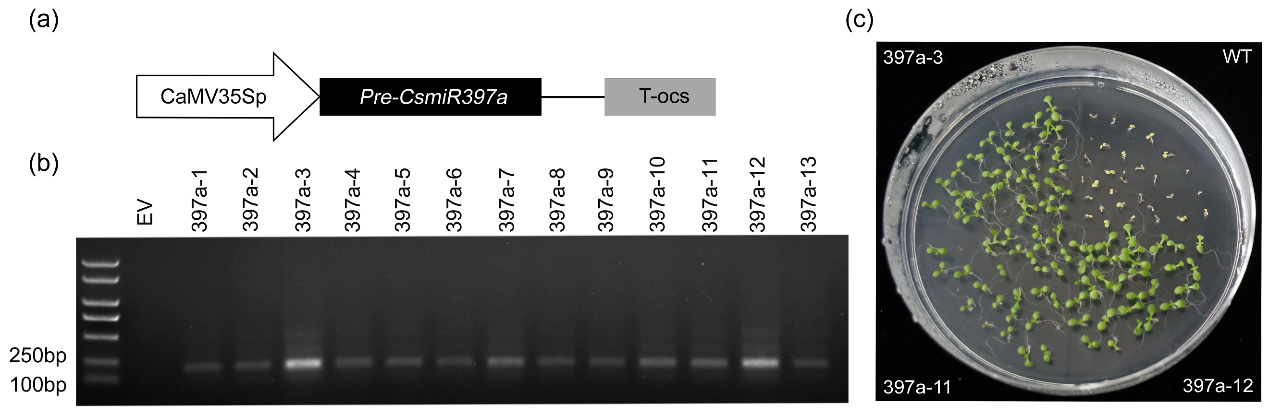


**Figure S3.** Identification of Arabidopsis overexpressing *CsmiR397a*. (a) Schematic of the construction of stably transformed *Arabidopsis thaliana*. (b) Semiquantitative results of *CsmiR397a* in different lines of Arabidopsis. (c) Identification of the T2 generation of homozygous Arabidopsis was performed on 1/2 MS medium with kanamycin.


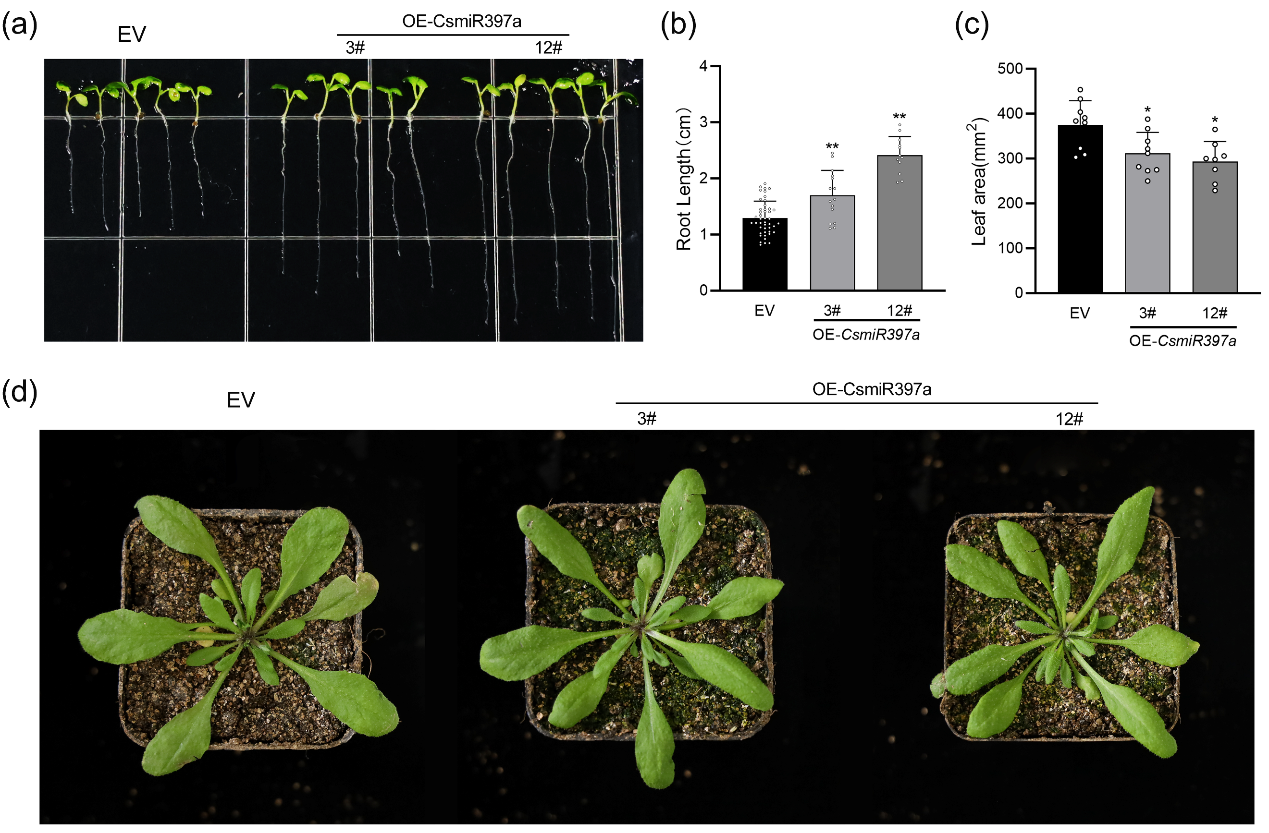


**Figure S4.** Phenotypes of transgenic Arabidopsis plants overexpressing the *CsmiR397a*. (a) Phenotypes of root length in transgenic Arabidopsis plants. (b, c) Statistical analysis of root length and leaf area in transgenic *Arabidopsis thaliana* (n = 9). (d) Phenotypes of transgenic Arabidopsis leaves. Each bar indicates the mean ± SD. Asterisks indicate significant differences from EV, ***p* < 0.01.


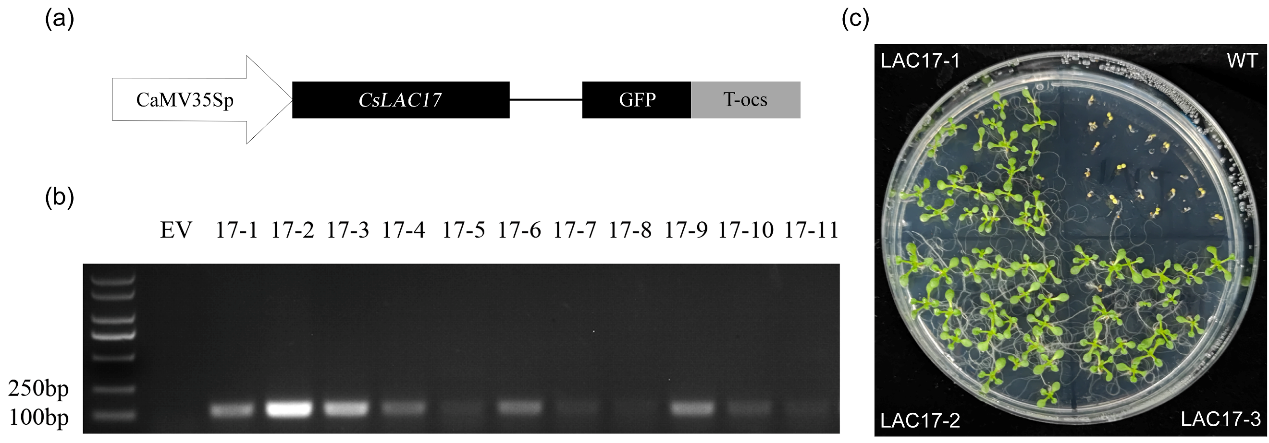


**Figure S5**. Identification of Arabidopsis-overexpressing *CsLAC17*. (a) Schematic of the construction of stably transformed Arabidopsis plants. (b) Semiquantitative results of *CsLAC17* in different lines of Arabidopsis. (c) Identification of the T2 generation of homozygous Arabidopsis was performed on 1/2 MS medium with kanamycin.


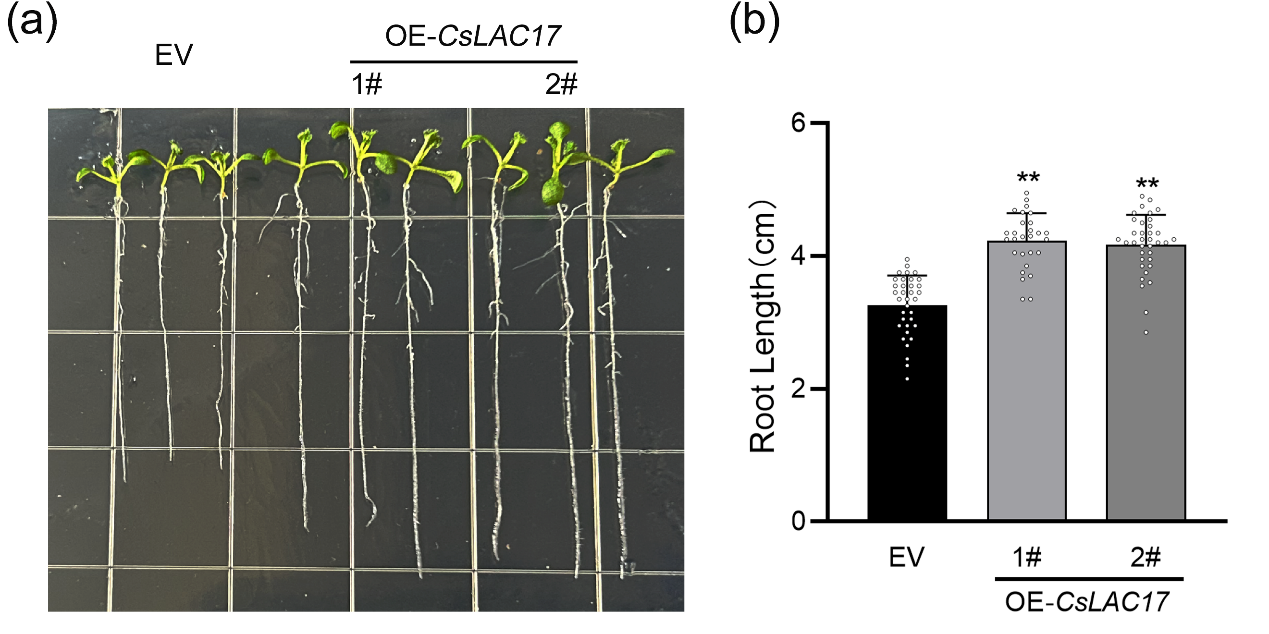


**Figure S6.** Phenotypes of transgenic Arabidopsis plants overexpressing the *CsLAC17*. (a) Phenotypes of root length in transgenic Arabidopsis plants. (b) Statistical analysis of root length in transgenic *Arabidopsis thaliana* (n > 20). Each bar indicates the mean ± SD. Asterisks indicate significant differences from EV, ***p* < 0.01.


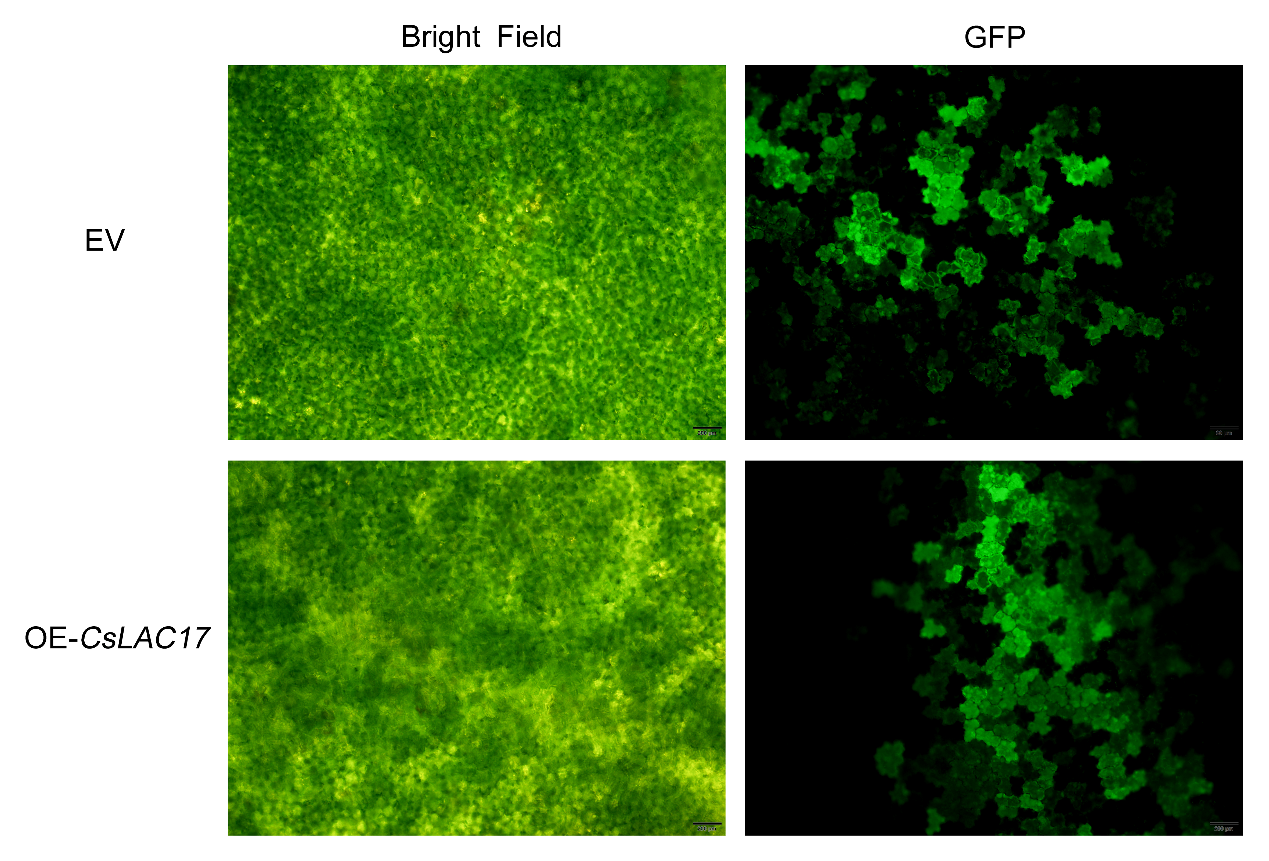


**Figure S7.** Green fluorescent signals of GFP alone (EV) and CsLAC17::GFP fusion (OE-*CsLAC17*) protein in leaf cells of tea plant after transient transformation.

**Table S1 Primers used in this work**

| **Name** | **Primer** | **Sequence (5’-3’)** | **Function** |
| --- | --- | --- | --- |
| *CsLAC17* | Forward | CATGGGTTCTTCTTCATTTATTCCATCTCC | Gene clone |
|  | Reverse | GCTCAACATGTAGGAAGATCGGAGG |  |
| *CsmiR397a* | Forward | TATTCTATGTACTTCATAGCCTTAT |  |
|  | Reverse | TAATAATACTAGAAGTTGGGCAGCA |  |
| *CsLAC17* | Forward | GCAAGCATTACAAGGCACTACAAGT | qPCR |
|  | Reverse | GATCGCCCTCTCTAGCAATAATCCG |  |
| *AtLAC2*  (AT2G29130) | Forward | ATGCAGCATCTGCAGGAA |  |
|  | Reverse | CTTCTCTTGCGGTAACTCTAGG |  |
| *AtLAC17*  (AT5G06390) | Forward | GTCGTACAACCGGTATTGTGCT |  |
|  | Reverse | TGTCTCTTACAATTTCCCGCTCT |  |
| *CsActin* | Forward | TAGAAACCCCAAGTACCCTCG |  |
|  | Reverse | TGCTTTCTTCGTCCCATCAG |  |
| *AtActin* | Forward | GTCGTACAACCGGTATTGTGCT |  |
|  | Reverse | TGTCTCTTACAATTTCCCGCTCT |  |
| *AtU6* | Forward | CGGGGACATCCGATAAAATT |  |
|  | Reverse | TTGGACCATTTCTCGATTTG |  |
| *Pre*-*CsmiR397a* | Forward | GAGTGCAGCGTTGATGAATCATCCCA |  |
|  | Reverse | provide by kit (Accurate, China) |  |
| *CsmiR397a* | Forward | TAATTGAGTGCAGCGTTGATG |  |
|  | Reverse | provide by kit (Accurate, China) |  |
| *CsmiR222* | Forward | TTTCCAAGACCACCCATGCCGA |  |
|  | Reverse | provide by kit (Accurate, China) |  |
| *35S*::*Pre-CsmiR397a* | Forward | GAGCTCGGTACCCGGGGATCCCTTAAAAACATAATTGAGTGCAGCG | Dual luciferase assay |
|  | Reverse | ATACGAACGAAAGCTCTGCAGGCCAAAAACATGATTGAGTGCA |  |
| *35S*::*CsLAC17-LUC* | Forward | TTTGGAGAGAACACGAAGCTTATGGGTTCTTCTTCATTTATTCCATC |  |
|  | Reverse | CGCTCTAGAACTAGTGGATCCCGACATGTAGGAAGATCGGAGG |  |
| *35S*::*mCsLAC17-LUC* | Forward | TTTGGAGAGAACACGAAGCTTATGGGTTCTTCTTCATTTATTCCATC |  |
|  | Reverse | CGCTCTAGAACTAGTGGATCCCGACATGTAGGAAGATCGGAGG |  |
| *35S*::*CsLAC17-GFP* | Forward | TTTGGAGAGAACACGAAGCTTATGGGTTCTTCTTCATTTATTCCATC | Subcellular localization and genetic transformation |
|  | Reverse | CGCTCTAGAACTAGTGGATCCCGACATGTAGGAAGATCGGAGG |  |
| adaptor sequence | GCUGAUGGCGAUGAAUGAACACUGCGUUUGCUGGCUUUGAUGAAA | | RLM-RACE for cleavage site validation of *CsmiR397a* |
| RACE outer primer | GCTGATGGCGATGAATGAACACTG | |  |
| RACE inner primer | CGCGGATCCGAACACTGCGTTTGCTGGCTTTGATG | |  |
| *CsLAC17* outer R | TGTTCAAACTGCGAAATGTGGAAGTG | |  |
| *CsLAC17* inner R | CGCCATTAAGAAAGCGGTGTTGTTGG | |  |
| *CsmiR397a-*agomir | sense: UAAUUGAGUGCAGCGUUGAUG  antisense: UCAACGCUGCACUCAAUUAUU | | miRNA overexpression |
| Negative control | UCACAACCUCCUAGAAAGAGUAGA | |  |
